# A high level of satisfaction after bicompartmental individualized knee arthroplasty with patient-specific implants and instruments

**DOI:** 10.1007/s00167-018-5155-4

**Published:** 2018-10-05

**Authors:** Takahiro Ogura, Kiet Le, Gergo Merkely, Tim Bryant, Tom Minas

**Affiliations:** 1 Sports Medicine Center, Funabashi Orthopaedic Hospital, 1-833 Hasama, Funabashi, Chiba 274-0822 Japan; 2 Newton–Wellesley Sports Medicine, 2000 Washington St., Blue Building, Suite 423, Newton, MA 02462 USA; 3 Department of Traumatology, Semmelweis University, Uzsoki u. 29, Budapest, 1145 Hungary; 4 Cartilage Repair Center, Paley Orthopedic and Spine Institute, St. Mary’s Hospital, 901 45th Street, Kimmel Building, West Palm Beach, FL 33407 USA

**Keywords:** Bicompartmental knee arthroplasty; osteoarthritis, Resurfacing, Customized individually made, Patient-specific implantation

## Abstract

**Purpose:**

Customized Individually Made (CIM) Bicompartmental Knee Arthroplasty (BKA) implants and three-dimensional printed customized instruments are available to fit to each patient’s unique anatomy, medial or lateral with patellofemoral. This study aimed to evaluate the clinical outcomes after CIM-BKA.

**Methods:**

Fifty-five patients [59 knees; average age, 51 years; standard deviation (SD), 6.8; range 37–65 years] who underwent CIM-BKA were evaluated over an average of 3.8-year follow-up (SD 1.6; range 1–6 years). Forty-one knees underwent BKA combined medial and patellofemoral replacement (BKA-MP) and 18 knees underwent BKA combined lateral with patellofemoral replacement (BKA-LP). Survival rates, the modified Cincinnati Knee Rating Scale, WOMAC, VAS, SF-36, a satisfaction survey, and radiographic evaluation were used to evaluate outcomes.

**Results:**

Overall, survival rates were 98% and 92% at 2 and 5 years, respectively. Of 56 knees (95%) that did not fail, all patient-reported functional scores significantly improved post-operatively (*P* < 0.01), regardless of the previous surgeries, with a high level of satisfaction (51/56 knees, 91%). Radiographically, all the femoral components fit perfectly and 56 knees (95%) of the tibial components fit with less than 2 mm of undercoverage or overhang. Three knees (5%) required the conversion to TKA and 17 knees (29%) required subsequent surgical procedures, of which multiply operated knees had higher rate than virgin knee [14/40 (35%) vs. 3/16 (19%)].

**Conclusion:**

CIM-BKA allowed precise fit of the components and provided a significant improvement post-operatively with a high level of satisfaction over short- to mid-term follow-up. This novel CIM-BKA is resurfacing, and does not require 10-mm faceted cuts, being only 3-mm-thick, which preserves bone stock for the future. It may be a promising option for relatively young active patients with bicompartmental osteoarthritis with a longer term follow-up being necessary.

**Level of evidence:**

IV.

## Introduction

Osteoarthritis (OA) of the knee is one of the most common diseases that cause pain and dysfunction [17] and affects many health outcomes [16]. For the treatment of OA, total knee arthroplasty (TKA) is the standard of care that provides high reproducibility of outcomes except in young patients where the failure rate is higher, and patient satisfaction and improvement are lower than older patients [10, 18, 26, 29]. Recently, a systematic review showed that, in patients under 65 years old, unicompartmental knee arthroplasty (UKA) provided higher functional outcomes but higher revision rates than TKA [14]. Therefore, treating OA in young patients is challenging.

For those who have localized arthritis rather than tricompartmental arthritis, however, sacrificing the other unaffected compartment and cruciate ligaments by performing TKA is concerning, as cruciates play an important role on load distribution, stability [30], and possibly proprioception [11]. Preservation of bone stock is desirable when considering future revision surgeries in a young active patient. A pattern of wear with bicompartmental OA is commonly observed in patients who undergo TKA. To manufacture a bicompartmental arthroplasty, a computer tomography (CT)-based, customized, patient-specific Bicompartmental Knee Arthroplasty (BKA) and printed nylon individualized instruments have been developed.Customized Individually Made (CIM) BKA implants (ConforMIS Inc, Burlington, MA) and three-dimensional printed customized surgical instruments are currently available to fit to each patient’s unique anatomy, medial and patellofemoral, or lateral and patellofemoral. Unique to this implant is that it resurfaces the 3 mm of cartilage on the subchondral bone with a 3-mm-thick implant and only removes a 3–5-mm anterior and posterior femoral cut to implant the prosthesis. Historically, a bicompartmental monolithic implant, made from a standard prosthesis less a condyle using standardized faceted 10 mm cuts, has been available in the past but taken off the market because of poor outcomes due to impingement and fit issues. These occurred at the patellofemoral native compartment junction as well as tibial baseplate fractures resulting in a high failure rate of up to 60% at a minimum follow-up of 54 months [8, 22]. Otherwise, modular “off-the-shelf” implants using unicondylar implants combined with a patellofemoral implant are required. However, controversy still exists regarding the functional outcomes when compared to TKA [23, 31]. To date, scarce data are available on the treatment of bicompartmental osteoarthritis. The outcomes of this novel resurfacing CIM-BKA have not been previously reported. The aim of this study was to evaluate the clinical outcomes and patient satisfaction of patients after CIM-BKA, previously unreported, over the short- to mid-term follow-up. The hypothesis in this study was that a significant improvement in pain and function with a high level of satisfaction would be provided after CIM-BKA while preserving the cruciates and bone stock.

## Materials and methods

A total of 55 patients underwent CIM-BKA (ConforMIS Inc) for symptomatic bicompartmental OA (combined medial or lateral with patellofemoral OA) between September 2010 and February 2016. A single surgeon performed all procedures. This cohort represented approximately 10% of the cases performed during this period for which TKAs were performed, average 100/year. Of this cohort, we evaluated all 55 patients (59 knees; average age, 51 years; SD 6.8; range 37–65) over an average of 3.8-year follow-up [standard deviation (SD) 1.6; range 1–6 years] as all had successfully completed more than 1 year of follow-up by the time of data analysis. There were 35 women and 24 men with an average body mass index (BMI) of 28.3 (SD 4.8; range 19.2–39.3 kg/m^2^). A total of 41 knees underwent BKA combined medial and patellofemoral replacement (BKA-MP), and 18 knees underwent BKA combined lateral and patellofemoral replacement (BKA-LP) (Table 1). Simultaneous bilateral procedure was performed in 1 patient. Indications for this surgery were bicompartmental joint disease including combined medial or lateral with patellofemoral OA. Contraindications to this surgery included tricompartmental OA, ligament instabilities, or knee deformities greater than 15°. Patients who had prior high tibial osteotomy (HTO) were not considered contraindication. Standing long-alignment radiographs to include hip/knee/ankle, standing anteroposterior (AP), Rosenberg, lateral radiographs, and skyline view were evaluated pre-operatively. In addition, either MRI or CT arthrography was performed pre-operatively to assess the whole joint before considering BKA.

**Table 1:** Patient demographics

	BKA-MP (*n* = 41 knees)	BKA-LP (*n* = 18 knees)	Overall (*n* = 59 knees)
Variables			
Age at surgery (years), mean ± SD (range)	51.6 ± 6.9 (41–65)	48.6 ± 6.4 (37–60)	50.7 ± 6.8 (37–65)
Gender, male/female, *n*	16/25	8/10	24/35
Right/left knee, *n*	22/19	10/8	32/27
Body mass index (kg/m^2^), mean ± SD (range)	28.7 ± 3.9 (20.1–39.3)	27.1 ± 6.4 (19.2–39.3)	28.2 ± 4.8 (19.2–39.3)

*BKA-MP* bicompartmental knee arthroplasty combined medial and patellofemoral replacement, *BKA-LP* bicompartmental knee arthroplasty combined lateral with patellofemoral replacement, *SD* standard deviation

Prior to the index surgery, 42 of all 59 knees (71%) had a total of 141 procedures (mean 3.4; SD 2.8; range 1–13) primarily including a total of 107 arthroscopic procedures (primarily partial meniscectomy and chondroplasty) in 39 knees (mean 2.7; SD 2.2; range 1–11) (Table 2). Pre-operative AP and lateral radiographs were scored in accordance with the K–L grade [13] to evaluate the progression of OA before the index surgery. Radiographic evaluation showed that 61% knees were grade 2, 29% were grade 3, and 10% were grade 4 based on the Kellgren–Lawrence (K–L) grading system.

**Table 2:** Number of knees with the previous surgical procedures

Procedure	Number of knees
A/S chondroplasty and meniscectomy	39
ACI total	8
ACI combined with osteotomy^a^	5
ACI alone	2
ACI combined with MAT	1
Osteotomy alone total	8
HTO	5
TTO	2
Extension femoral osteotomy	1
ACLR	4
Osteochondral allograft transplantation	2
OATS combined with TTO	1
Unicompartmental knee replacement	1
Others	4
No previous procedures	17

The 141 previous procedures were performed among 42 knees (71%). Some patients had more than one procedure

Others included removal of hardware in 1 and drainage of wound infection in 1

*A/S* arthroscopic,* TTO* tibial tubercle osteotomy,* HTO* high tibial osteotomy,* ACI* autologous chondrocyte implantation,* MAT* meniscal allograft transplantation,* ACLR* anterior cruciate ligament reconstruction,* OATS* osteochondral autologous transplantation

^a^Osteotomy included tibial tubercle osteotomy [TTO] in 2, HTO in 2, and TTO/distal femoral osteotomy [DFO] in 1

### Operative technique

**Fig. 1:**
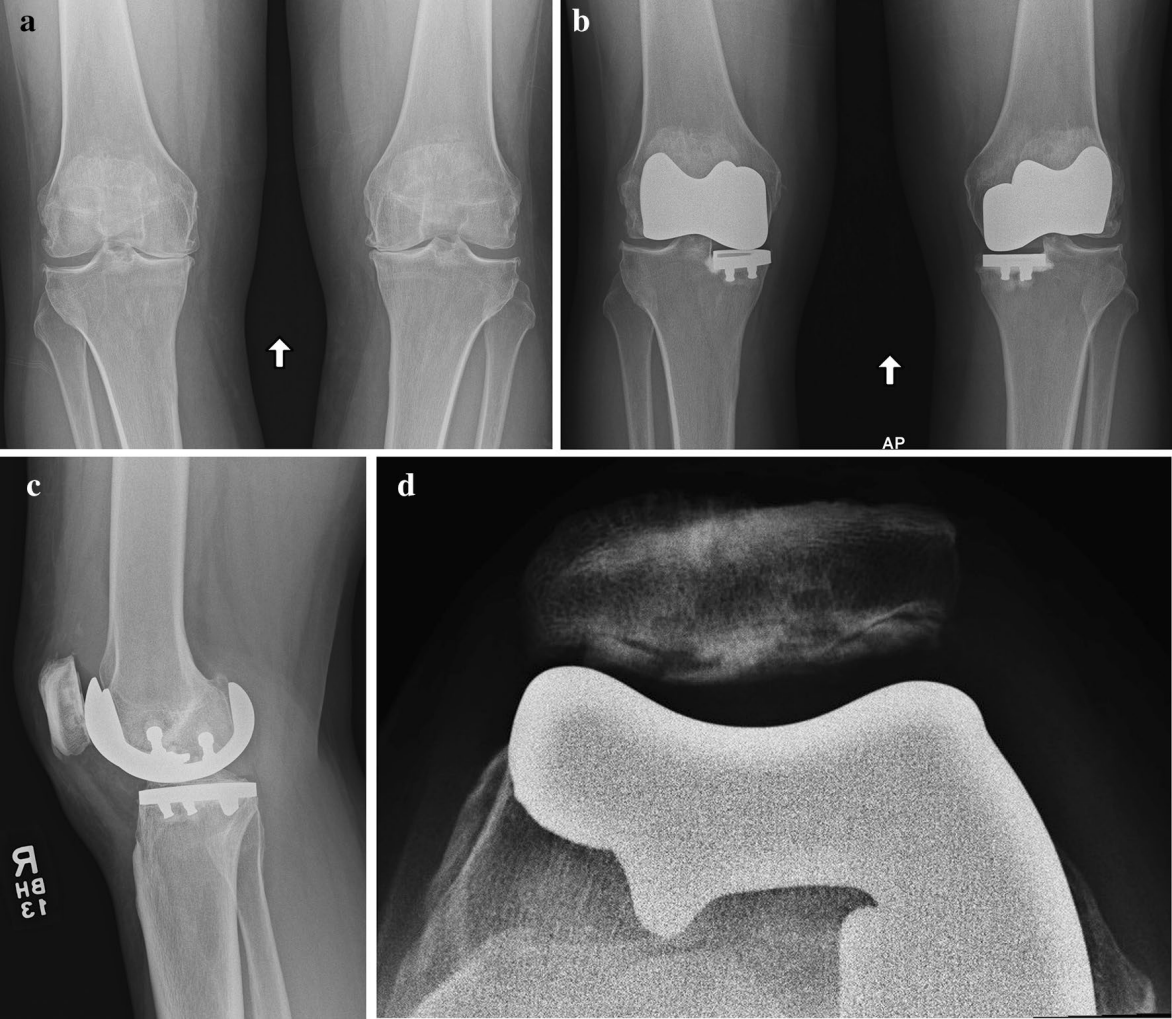
Bicompartmental knee arthroplasty (BKA). Pre-operative bilateral anteroposterior radiograph of a 44-year-old woman with disabling anterior and medial joint pain (**a**). Post-operative bilateral anteroposterior (**b**), lateral (**c**), and skyline (**d**) radiographs of a patient after bilateral BKA combined medial and patellofemoral replacement

Based on the CT imaging of the affected lower extremity (from hip to ankle), the patient-specific instruments and implants are manufactured. The surgical techniques have been described elsewhere in detail [20, 27]. Briefly, a medial or lateral parapatellar arthrotomy is performed dependent on the compartments treated. The patella is resurfaced in the standard fashion for TKA with an off-the-shelf polyethylene button. The remaining cartilage in the affected trochlea and femoral condylar compartments is removed using ring curettes and a #10-blade. Then, the osteophytes are removed to the native cortex (confirmed with the pre-operatively planned ‘iView^TM’^ protocol.) The knee joint is balanced in extension and flexion using the patient-specific “balancing chip” jigs, with both anterior and posterior cruciate ligaments being preserved. Tibial bone cuts are performed with the balancing chip jigs being linked to the tibial cutting guide. No distal bone was removed from the femur as the implants fits directly on the subchondral bone plate. Thin anterior and posterior cuts of the anterior trochlea and posterior femoral condyle allow the placement of the trial nylon printed implant. The femoral subchondral bone is prepared for cement penetration with multiple 2-mm drills holes with a step drill to improve cement inter-digitation. The final cobalt-chrome patient-specific femoral implant is cemented in place after the tibial component is cemented with a 5- or 7-mm polyethylene trial insert. The final polyethylene tibial component is inserted into the tibial tray (Fig. 1). The wound is closed in layers with a soft-compressive dressing, over a drain that is removed the next day.

### Post-operative course

Post-operatively, patients were allowed full weight-bearing and range of motion on the next day of the surgery. The standard thrombosis prophylaxis using 325 mg ASA bid for 3 weeks was utilized. Functional rehabilitation was performed in the same manner as a conventional TKA.

### Survival analysis and clinical outcomes assessment

**Fig. 2:**
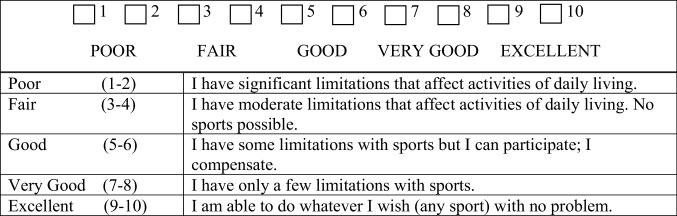
Modified Cincinnati Knee Rating scales

Treatment failure was defined as conversion to TKA or removal of implant. Unicompartmental knee replacement for the opposite compartment was not considered as treatment failure as per a recent study [1]. Survival analysis was performed using the Kaplan–Meier method, with treatment failure as the endpoint. The modified activity Cincinnati Knee Rating Scale [19], the Western Ontario and McMaster Universities Osteoarthritis Index (WOMAC) [2], the Visual Analog Scale (VAS), and Short Form-36 (SF-36) [3] were used to evaluate clinical outcomes. Patients also self-reported knee function and satisfaction. The original Cincinnati Knee Rating Scale is based on a 0–100 continuous scale [21], whereas the modified Cincinnati Knee Rating Scale is based on a 1–10 categorized scale, with a two-point change being considered clinically meaningful (Fig. 2) [4]. Scores were collected pre-operatively and at yearly intervals post-operatively during an office visit or by mailed questionnaire. Additional analyses were performed on the two major cohorts, “virgin” knees or multiply operated knees to see if there was a difference in baseline characteristic and functional outcomes.

### Post-operative radiographic evaluation

For analyzing the undercoverage or overhang of tibia tray and femoral component, post-operative AP radiographs were reviewed. Undercoverage was defined as the distance between the implant and the edge of the tibial plateau/femoral condyle. Overhang was defined as the distance between the edge of the tibia/femoral condyle and the edge of the tibial tray/femoral component. Undercoverage or overhang of the femoral component or tibial tray at the joint line was measured on a digital picture archiving and communication system (PACS) on calibrated DICOM radiographs.

This study was approved by the Institutional Review Board of Cartilage Repair Center, Brigham and Women’s Hospital, Harvard Medical School, Boston, Massachusetts (2007P000470), and written consent was obtained from all patients.

### Statistical analysis

All statistical analyses were performed with Stata (version 13; Statacorp LP, College Station, TX). Kaplan–Meier curves were used for the survival analyses. The Wilcoxon signed-rank test was used to compare differences in functional scores (obtained from the modified Cincinnati, VAS, WOMAC, and SF-36) between the two time points (pre-operatively and at the final follow-up). Mann–Whitney *U* tests were used to compare the improvement in scores between different groups. The level of significance was set *a priori* at *p* < 0.05.

No sample size calculation was performed before conducting this study, because all patients who met the inclusion criteria were included. A post hoc power analysis was conducted using G*Power, version 3.1. A post hoc power analysis revealed a power of greater than 80% regarding all the functional scores except for WOMAC stiffness and WOMAC function after BKA-MP, which indicated that sample size was adequate to provide statistical significance to most of the observed differences.

Radiological measurements were performed twice by the same observer. The intra-observer reliability of these measurements was evaluated by an intraclass correlation coefficient. The intra-observer reliability was 0.975.

## Results

### Survival analysis

**Fig. 3:**
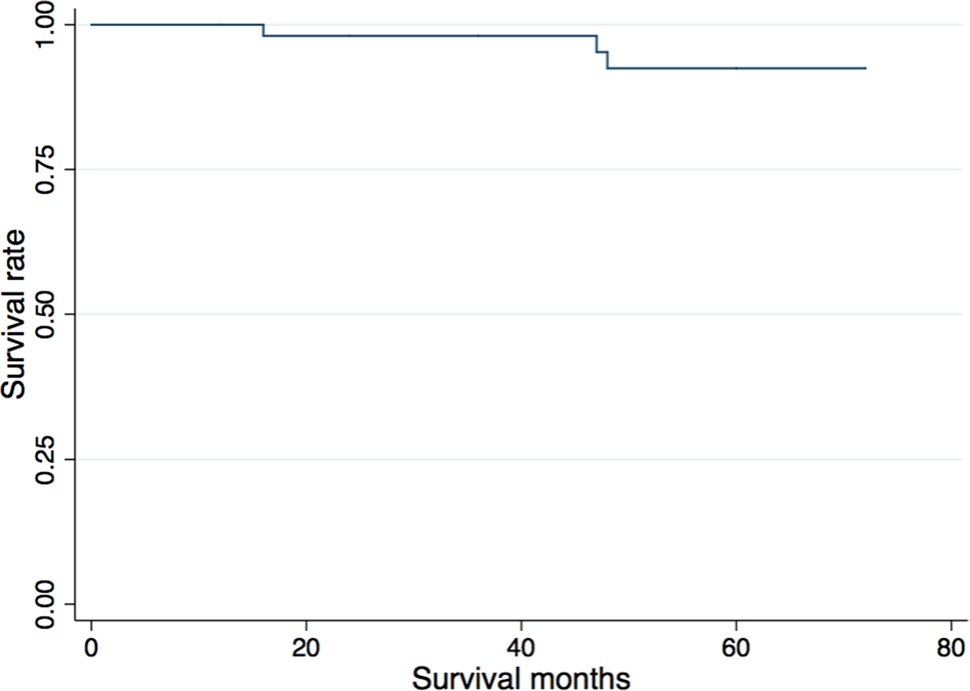
Kaplan–Meier survival curve. Overall (*n* = 59 knees), survival rates were 98% and 92% at 2 and 5 years, respectively

Among all 59 knees, 3 knees (5%) were considered to be failure (2 in BKA-MP and 1 in BKA-LP) at an average of 3.1 years post-operatively. Overall, survival rates were 98% and 92% at 2 and 5 years, respectively (Fig. 3).

### Functional outcomes

Of 52 patients (56 knees) who did not require conversion to TKA, all patient-reported functional scores were clinically meaningfully and significantly improved post-operatively. Of 39 knees successfully treated with BKA-MP, all functional scores except WOMAC stiffness and WOMAC function significantly improved post-operatively. Of 17 knees successfully treated with BKA-LP, all functional scores significantly improved post-operatively (Table 3).

**Table 3:** Pre-operative and final follow-up clinical outcomes in successfully treated knees with BKA-MP (*n* = 39 knees) and BKA-LP (*n* = 17 knees)

Rating system	BKA-MP (*n* = 39)			BKA-LP (*n* = 17)		
	Pre-operative	Final follow-up	*P* value	Pre-operative	Final follow-up	*P* value
Modified Cincinnati	1.9 ± 1.0	6.1 ± 1.9	< 0.001	1.9 ± 0.8	6.4 ± 2.0	< 0.001
VAS	6.3 ± 2.2	2.1 ± 1.4	< 0.001	5.5 ± 1.7	1.5 ± 1.3	< 0.001
WOMAC total	36.3 ± 16.9	28.2 ± 15.9	0.0092	36.6 ± 16.5	19.9 ± 13.7	0.0018
WOMAC—pain	8.3 ± 3.8	5.4 ± 3.7	< 0.001	8.1 ± 3.6	3.6 ± 2.8	< 0.001
WOMAC—stiffness	3.7 ± 1.7	3 ± 1.4	ns	3.5 ± 1.9	2.2 ± 1.6	0.0129
WOMAC—function	24.3 ± 12.5	20 ± 11.6	ns	25.1 ± 11.6	14.1 ± 9.7	0.0036
SF-36—PCS	38.6 ± 7.6	47.8 ± 8.4	< 0.001	38.5 ± 6.6	47.2 ± 10.4	0.0245
SF-36—MCS	40.5 ± 6.5	52.6 ± 9.9	< 0.001	40.5 ± 7.2	51.7 ± 10.6	0.0031

*BKA-MP* bicompartmental knee arthroplasty combined medial and patellofemoral replacement, *BKA-LP* bicompartmental knee arthroplasty combined lateral with patellofemoral replacement, *VAS* Visual Analog Scale, *WOMAC* Western Ontario and McMaster Universities Osteoarthritis Index SF-36, Short Form-36, *MCS* mental component score, *PCS* physical component score, *ns* not significant

### Patient satisfaction survey

Of patients with retained implants, 91% of the patients rated their knees as good or excellent, and answered that they were satisfied their knee surgery at the latest follow-up (Table 4).

**Table 4:** Satisfaction with the procedure at final follow-up (in the 56 successful knees)

Question	BKA-MP (*n* = 39 knees)	BKA-LP (*n* = 17 knees)	Overall (*n* = 56 knees)
What is your overall satisfaction level with the joint surgery?			
Satisfied	35 (90%)	16 (94%)	51 (91%)
Neutral	2 (5%)	0 (0%)	2 (4%)
Dissatisfied	2 (5%)	1 (6%)	3 (5%)
How would you rate the results of your joint surgery?			
Good/excellent	36 (92%)	15 (88%)	51 (91%)
Fair	2 (5%)	2 (12%)	4 (7%)
Poor	1 (3%)	0	1 (2%)
If you could go back in time and make the decision again, would you choose to undergo your joint surgery?			
Yes	36 (92%)	16 (94%)	52 (96%)
Uncertain	2 (5%)	0	2 (2%)
No	1 (3%)	1 (6%)	2 (2%)

*BKA-MP* bicompartmental knee arthroplasty combined medial and patellofemoral replacement, *BKA-LP* bicompartmental knee arthroplasty combined lateral with patellofemoral replacement

### Radiographic evaluation

In all cases, radiographic evaluation demonstrated an ideal and perfect fit of the femoral components. For the tibial components, 95% of the components fit within less than 2 mm of undercoverage or overhang (including 66% being fitted perfectly). No tibial components mismatched greater than 3 mm (Table 5).

**Table 5:** Post-operative radiographic evaluation

	Femoral component	Tibial component (%)
2–3 mm undercoverage	0	2 (3.4)
1–2 mm undercoverage	0	4 (6.8)
0–1 mm undercoverage	0	3 (5.1)
Perfect matching	59 (100%)	39 (66.1)
0–1 mm overhang	0	6 (10.2)
1–2 mm overhang	0	4 (6.8)
2–3 mm overhang	0	1 (1.7)

### Subanalysis: “virgin” knees vs. “multiply operated” knees

Of 41 patients who underwent BKA-MP, 13 patients constituted the cohort of the “virgin” knees and 28 patients constituted the cohort of the “multiply operated” knees. Patient age at the time of surgery was significantly older in the “Virgin” than “multiply operated” knees (*P* = 0.0247). The other variables showed no significant difference between the two cohorts. Of 18 patients who underwent BKA-LP, 4 patients constituted the cohorts of “virgin” knees and 14 patients constituted the cohorts of “multiply operated” knees. There was no significant difference in baseline characteristics between the two cohorts (Table 6).

**Table 6:** Patient demographics (virgin vs. multiply operated knee)

	Virgin knee (*n* = 17 knees)	Multiply operated knee (*n* = 42 knees)	*P* value
Variables			
BKA-MP	*n* = 13	*n* = 28	
Age at surgery (years), Mean ± SD (range)	55.2 ± 5.8 (45–65)	50 ± 6.8 (41–63)	0.0247
Gender, male/female, *n*	6/7	10/18	ns
Right/left knee, *n*	7/6	15/13	ns
Body mass index (kg/m^2^), mean ± SD (range)	28 ± 3.1 (22.2–33)	29 ± 4.2 (20.1–39.3)	ns
Previous surgeries, mean ± SD (range)	–	3.2 ± 2.8 (1–13)	–
BKA-LP	*n* = 4	*n* = 14	
Age at surgery (years), mean ± SD (range)	53 ± 3.2 (50–57)	47.4 ± 6.6 (37–60)	ns
Gender, male/female, *n*	0/4	8/6	ns
Right/left knee, *n*	3/1	7/7	ns
Body mass index (kg/m^2^), mean ± SD (range)	20.5 ± 1.7 (19.2–22.9)	29 ± 6.0 (20.7–39.3)	0.0137
Previous surgeries, mean ± SD (range)	–	3.6 ± 2.9 (1–10)	–

*BKA-MP* bicompartmental knee arthroplasty combined medial and patellofemoral replacement, *BKA-LP* bicompartmental knee arthroplasty combined lateral with patellofemoral replacement, *ns* not significant

Pre- and post-operative functional scores were compared for the two cohorts (Tables 7, 8). Of patients who underwent BKA-MP, pre-operative scores of VAS, WOMAC total, WOMAC pain, and WOMAC function were significantly lower in the “virgin” knees than the “multiply operated” knees. There was no significant difference in all post-operative scores between those cohorts. Of those who underwent BKA-LP, there was no significant difference in all pre- and post-operative scores between those cohorts.

**Table 7:** Post-operative functional scores in BKA-MP (virgin vs. multiply)

BKA-MP	Virgin knee (*n* = 12)	Multiply operated knee (*n* = 27)	*P* value
Modified Cincinnati			
Pre	2.1 ± 0.8	1.9 ± 1.1	ns
Post	6.3 ± 2.2	6.0 ± 1.8	ns
VAS			
Pre	5.3 ± 2.4	6.9 ± 1.9	0.0377
Post	1.4 ± 0.8	2.3 ± 1.6	ns
WOMAC total			
Pre	26.9 ± 10.5	40.5 ± 17.7	0.0101
Post	26 ± 12.0	29.1 ± 17.2	ns
WOMAC pain			
Pre	5.9 ± 2.4	9.4 ± 3.8	0.0027
Post	5.2 ± 2.1	5.5 ± 3.8	ns
WOMAC stiffness			
Pre	3.3 ± 0.9	3.9 ± 2.0	ns
Post	2.9 ± 0.9	3.0 ± 1.6	ns
WOMAC function			
Pre	17.7 ± 8.2	27.2 ± 13.1	0.0280
Post	17.9 ± 9.3	20.5 ± 12.1	ns
SF-36 PCS			
Pre	38.7 ± 6.3	38.6 ± 8.2	ns
Post	49.3 ± 8.4	47.2 ± 8.4	ns
SF-36 MCS			
Pre	41.8 ± 5.3	39.9 ± 7.0	ns
Post	55.3 ± 7.2	51.4 ± 10.7	ns

*BKA-MP* bicompartmental knee arthroplasty combined medial and patellofemoral replacement, *VAS* Visual Analog Scale, *WOMAC* Western Ontario and McMaster Universities Osteoarthritis Index SF-36, Short Form-36, *MCS* mental component score, *PCS* physical component score, *ns* not significant

**Table 8:** Post-operative functional scores in BKA-LP (virgin vs. multiply)

BKA-LP	Virgin knee (*n* = 4)	Multiply operated knee (*n* = 13)	*P* value
Modified Cincinnati			
Pre	2 ± 0	1.8 ± 0.9	ns
Post	7.8 ± 1.7	6 ± 1.9	ns
VAS			
Pre	5.8 ± 1.9	5.5 ± 1.7	ns
Post	0.8 ± 1.0	1.7 ± 1.4	ns
WOMAC total			
Pre	34.5 ± 3.4	37.3 ± 18.9	ns
Post	23.5 ± 9.8	18.8 ± 14.8	ns
WOMAC pain			
Pre	7.5 ± 1.3	8.3 ± 4.1	ns
Post	4 ± 2.7	3.5 ± 2.9	ns
WOMAC stiffness			
Pre	3 ± 0.82	3.6 ± 2.1	ns
Post	2.3 ± 1.7	2.2 ± 1.6	ns
WOMAC function			
Pre	24 ± 3.4	25.4 ± 13.2	ns
Post	17.5 ± 5.7	13.1 ± 10.6	ns
SF-36 PCS			
Pre	33.7 ± 7.8	40.0 ± 5.7	ns
Post	48.1 ± 13.6	46.9 ± 9.9	ns
SF-36 MCS			
Pre	38.5 ± 6.4	41.2 ± 7.6	ns
Post	56.2 ± 4.6	50.3 ± 11.7	ns

*BKA-LP* bicompartmental knee arthroplasty combined lateral with patellofemoral replacement, *VAS* Visual Analog Scale, *WOMAC* Western Ontario and McMaster Universities Osteoarthritis Index SF-36, Short Form-36, *MCS* mental component score, *PCS* physical component score, *ns* not significant

### Subsequent surgical procedures (SSPs)

Other than these three failed knees, 17 knees (30%) required SSPs (Table 9). Of the 16 “virgin” knees who did not have the previous surgery before the index surgery, 3 knees (3/16, 18.8%) required SSPs, whereas, of those with prior multiple surgeries, 14 knees (14/40, 35%) required SSPs. One patient had distal femoral extension osteotomy with an allograft medial collateral ligament reconstruction (MCLR) in 1 at 2 years to obtain hyperextension of 15°, which he had before BKA and neutral alignment to resolve his inward thrust after BKA. This patient had polio in the Middle East and had Grade 2/5 extensor mechanism weakness requiring hyperextension to lock his knee and walk without assistive device. Other complications including deep venous thrombosis or wound infection were not observed. No patient required any blood transfusion or blood products.

**Table 9:** Subsequent surgical procedures

Procedures	Knees, *n*	Timing post-operatively, years
A/S synovectomy	13	1.4 ± 1.1 (range 0.2–3.6)
A/S meniscectomy	2	0.8 and 3.2
UKR	2	0.9 and 1.9
Osteotomy with MCLR	1	2

*A/S* arthroscopic, *UKR* unicompartmental knee replacement, *MCLR* medial collateral ligament reconstruction

## Discussion

The most important finding of the present study was that CIM-BKA provides very high level of satisfaction with a significant improvement in pain, function, and mental health in young arthritic patients over a short- to mid-term follow-up by allowing precise fit of femoral components with 66% of tibial components being precisely fit and 95% being less than 2 mm of undercoverage or overhang.

Although substantial improvement in clinical outcomes is reported after TKA in young patients, increased risks of failure rates, less improvement, and dissatisfaction after TKA in those young and early OA patients have been shown [10, 26]. Moreover, a recent systematic review showed that, in patients under 65 years old, unicompartmental knee arthroplasty (UKA) provided higher functional outcomes but higher revision rates than TKA [14]. In young and early OA patients who are difficult to treat, a high level of satisfaction observed in this study, 91%, could be due to restoration of anatomical ‘J’ curves leading to more normal kinematics [30] and preservation of ACL and PCL, leading to a more normal proprioception provided by a customized implant based on each patient’s individual anatomy. In addition, the modified activity Cincinnati score showed a mean of 6.1 post-operatively, which indicated that patients were able to participate in sports activities. This achievement might contribute to high level of satisfaction, as well.

The subanalysis revealed that some of the pre-operative functional scores were significantly lower in the multiply operated knees than the “Virgin” knees. Nevertheless, the post-operative functional scores in both cohorts were comparable. Several studies reported the negative effects of the previous surgeries on the outcomes after TKR [10, 24, 25]. However, our results indicate that the individualized BKA may also be encouraging for those who had multiple previous surgeries unlike other prior studies looking at TKA.

Relatively high rate of SSPs was observed, which might be due to the baseline nature of young multiply operated knees before the index surgery. In fact, the rate of SSPs in the patients who did not have the previous surgeries was lower (18.8%) compared to those who had multiple prior surgeries (35%). The previous surgeries in our study primarily included arthroscopic procedures and HTO. Similar observation was found in a previous study that showed significantly higher post-operative complications (30%) in patients who had the previous arthroscopic debridement than those who did not (10%) [24].

Three patients were considered as a treatment failure and converted to TKA. Of those, no patients required stems, wedges, or PS components, which we believe support the philosophy of bone preservation and cruciate preservation. Similar failure rates were reported in patients after UKA [9, 28]. The first patient failed due to loosening of a tibial tray at 4 years, which is a common cause for revision surgery after UKA [1]. The second patient failed due to progression of the opposite compartment, also a common problem with UKA [1]. Because the BKA implant was well fixed, the treatment options included unicompartmental arthroplasty with retaining BKA implant rather than TKA. However, the patient underwent TKA due to her preference. The third patient, an obese, BMI 41 kg/m^2^, diabetic male developed a late infection after an initial excellent post-operative course and outcome, requiring a staged explant with antibiotic cement spacer and a primary CR TKA. Two patients who required unicompartmental arthroplasty on the opposite compartment after the CIM-BKA were not considered as a treatment failure as the BKA was well fixed and a lateral UKA dealt with the progression of OA-preserving bone stock and both cruciates. Clarifying a risk factor for the progression of OA on the remaining compartment will be necessary to avoid multiple replacement surgery. Most recently, a multi-center study showed the implant survivor rate after Oxford UKA was 90% at 10 years using the same approach, not including unicompartmantal arthroplasty on the opposite compartment as a failure [1]. The survival rates in this study (98% and 92% at 2 and 5 years, respectively) were comparable, although a long-term follow-up is still necessary to confirm the durability of this unique implant.

Although BKA theoretically can provide better functional outcomes due to the preservation of ligaments, proprioception, and distribution of joint loading, controversy still exists regarding the superiority of BKA over TKA. Our study showed that patient-specific BKA is promising over a short- to mid-term follow-up without any concerns about the gap between transitional edge of the trochlear component and the proximal edge of the native femoral articular surface as it is monolithic and designed to “dive” 5 mm deep to the subchondral bone based on the planning CT scan. In addition, it can offer an option for lateral OA combined with PF OA which only could have been manageable with an unlinked implant using implants manufactured basically for medial tibiofemoral OA instead of for lateral tibiofemoral OA.

A previously reported “off-the-shelf” monolithic implant required surgeons to implant the definitive implantation on the coronal plane to best resurface the medial and PF compartments [8, 22]. Conversely, a patient-customized implant can cover each patient-specific resurfacing precisely as demonstrated by our X-ray analyses fit of the femoral implant in all operated knees and precise fit of the tibial implant in 66% of all patients. Although 34% of patients did not show a precise fit of the tibial implant, the findings that 95% of all patients showed less than 2 mm of undercoverage or overhang are encouraging. The amount of osteophytes that are needed to be removed during the surgery depends on the surgeon, although the I-view protocol suggests the estimated osteophytes that should be removed. Thus, it might be difficult to determine the precise volume of osteophytes that should be removed during the surgery, which might have resulted in not perfectly precise fit in some cases. The previous studies have shown that an overhang of ≥ 3 mm in femoral and tibial component coverage was associated with an increased risk for knee pain after TKA [15] and UKA [5]. In the present study, no patients had undercoverage or overhang greater than 3 mm on a digital radiograph. Another human cadaveric study demonstrated overhang of the tibial tray greater than 2 mm can load and impinge on the medial collateral ligament and possibly cause knee pain [12]. Thus, a precise fit of the implant may be crucial to preventing soft-tissue irritation with pain and CIM- BKA is encouraging.

There were several limitations in this study. First, this is a case series with no control groups and not a randomized clinical trial against TKA or BKA with linked uni and PF replacements. However, thankfully, it is very difficult to find patients who have had multiple previous surgeries before prosthetic arthroplasty. Second, this study is short- to mid-term follow-up study. Thus, longer follow-up study will be needed to confirm the durability of this implant and whether patient functional outcomes and high level of satisfaction are maintained. Third, we measured overhang and undercoverage using digital radiographs which are not as accurate as measurement based on CT. However, prior studies have used post-operative radiographs to evaluate the fit of the implant [6, 7]. Fourth, we did not evaluate pre- and post-operative range of motion of the knee. This evaluation will be necessary in the long-term follow-up study. Finally, we did not include patients who had UKR on the opposite compartment. However, our definition for failure was consistent with the previous study [1].

## Conclusion

This customized resurfacing implant provided a precise fit of femoral components and a precise fit of 66% of tibial components with 95% of tibias being less than 2 mm of undercoverage or overhang. This novel resurfacing implant provided a high level of patient satisfaction with significant improvement in pain, function, and mental health over the short- to mid-term follow-up for the treatment of bicompartmental OA in young arthritic patients, whose average age was 51 in this study. Although there was a relatively high rate of SSPs observed particularly in patients with multiply operated knees, CIM-BKA allows the surgeon to achieve individualized optimal implantation and may be a satisfactory option for bicompartmental OA. A longer term follow-up is necessary to determine its role in the knee arthroplasty armamentarium.

## Abbreviations

OA: Osteoarthritis
BKA: Bicompartmental knee arthroplasty
CIM: Customized individually made
TKA: Total knee arthroplasty
